# Raman spectroscopy and mass spectrometry identifies a unique group of epidermal lipids in active discoid lupus erythematosus

**DOI:** 10.1038/s41598-023-43331-3

**Published:** 2023-09-30

**Authors:** Hannah U Holtkamp, Claude Aguergaray, Kalita Prangnell, Christopher Pook, Satya Amirapu, Angus Grey, Cather Simpson, Michel Nieuwoudt, Paul Jarrett

**Affiliations:** 1 The Photon Factory, The University of Auckland, Auckland, New Zealand; 2 School of Chemical Sciences, The University of Auckland, Auckland, New Zealand; 3 The Dodd Walls Centre for Photonic and Quantum Technologies, Dunedin, New Zealand; 4 Department of Physics, The University of Auckland, Auckland, New Zealand; 5 Liggins Institute, The University of Auckland, Auckland, New Zealand; 6 Department of Anatomy and Medical Imaging, The University of Auckland, Auckland, New Zealand; 7 Department of Physiology, The University of Auckland, Auckland, New Zealand; 8 The MacDiarmid Institute for Advanced Materials and Nanotechnology, Wellington, New Zealand; 9 Department of Dermatology, Middlemore Hospital, Auckland, New Zealand; 10 Department of Medicine, The University of Auckland, Auckland, New Zealand

**Keywords:** Cell biology, Biomarkers, Diseases, Optics and photonics

## Abstract

Discoid lupus erythematosus (DLE) is the most common form of cutaneous lupus^1^. It can cause permanent scarring. The pathophysiology of is not fully understood. Plasmacytoid dendritic cells are found in close association with apoptotic keratinocytes inferring close cellular signalling. Matrix Associated Laser Desorption Ionisation (MALDI) combined with Fourier Transform Ion Cyclotron Resonance Mass Spectrometry (FT-ICR-MS) is an exquisitely sensitive combination to examine disease processes at the cellular and molecular level. Active areas of discoid lupus erythematosus were compared with normal perilesional skin using MALDI combined with FT-ICR-MS. A unique set of biomarkers, including epidermal lipids is identified in active discoid lupus. These were assigned as sphingomyelins, phospholipids and ceramides. Additionally, increased levels of proteins from the keratin, and small proline rich family, and aromatic amino acids (tryptophan, phenylalanine, and tyrosine) in the epidermis are observed. These techniques, applied to punch biopsies of the skin, have shown a distinctive lipid profile of active discoid lupus. This profile may indicate specific lipid signalling pathways. Lipid rich microdomains (known as lipid rafts) are involved in cell signalling and lipid abnormalities have been described with systemic lupus erythematosus which correlate with disease activity.

## Introduction

Discoid lupus erythematosus (DLE) is the most common form of cutaneous lupus^1^. It causes permanent scarring, scarring alopecia, and hyper- or hypo-pigmentary change which is distressing for patients with skin of colour. DLE causes significant detrimental psychological impairment, and the extent of cutaneous involvement correlates with the Dermatology Life Quality Index^2,3^. The pathophysiology of DLE is not fully understood.

Histologically the findings of DLE depend on timing of the disease. The epidermis, dermis and hair follicles are involved. There is an interface dermatitis which includes hair follicles and a perivascular and periappendageal lymphocytic infiltrate in the upper and lower dermis, epidermal vacuolisation and apoptotic basal cells accompanied with marked hyperkeratosis. The basement membrane may become thickened and dermal mucin can be present. Evolving further, the inflammatory infiltrate subsides with dermal fibrosis^4^.

Plasmacytoid dendritic cells are found in close association with apoptotic keratinocytes in cutaneous lupus and these cells are an important source of Type-1 interferon which is a key cytokine in the pathogenesis and activity of cutaneous lupus^5^.

Scarring is a feature of DLE. Transforming growth factor β dependent pathways inducing fibrosis are important^6^. MicroRNAs are a non-coding RNA molecule that are important in regulating gene expression. Over expression of the microRNA miR-31, derived from keratinocytes, and miR-485-3p, derived from leukocytes, are important regulators of the pathogenesis of DLE through the production of inflammatory cytokines, including transforming growth factor β, and by promoting fibrosis^7^.

Raman spectroscopy is used in vivo and in vitro to examine the molecular composition of tissues with resolution possible at single cell level^8^. It is non-invasive, non-destructive and minimal sample preparation is required. Laser interactions with tissue scatter back light of different energies (inelastic scatter) which are recorded as Raman frequencies. These are distinctive for different molecules. The resulting Raman spectrum is an optical fingerprint of the chemical composition of the tissue. In combination with confocal microscopy Raman spectroscopy can be applied in imaging of entire tissue sections, creating a chemical image of the functional biomolecules. This is also termed hyperspectral imaging because of the large arrays of spectral information collected.

Mass spectrometry can characterise intact biomolecules by soft ionisation techniques such as Matrix Associated Laser Desorption Ionisation (MALDI). The sample is coated in a layer of crystalline matrix, and as the laser is pulsed on the surface, energy is transferred to the matrix surface then to the sample to generate intact, charged molecules (ions). The charge transfer is essential because it allows manipulation of the ions by external electric and magnetic fields for separation and measurement according to their mass-to-charge ratio (m/z). Using Fourier Transform Ion Cyclotron Resonance Mass Spectrometry (FT-ICR-MS) the ions produced are placed in a cyclical orbit and the frequency of their rotations are calculated. A small ion carrying the same charge as a large ion, travels faster and has a higher frequency leading to separation before detector measurement. FT-ICR-MS analysis provides high resolution mass accuracy. MALDI with FT-ICR-MS can profile different biomolecular species from intact sectioned tissues, determining their relative abundance and location in two dimensions^9^.

There are biomolecular differences between diseased and healthy tissue which can be detected by MALDI which is a highly sensitive technique. MALDI can identify individual biomolecules, particularly lipids. MALDI imaging using FT-ICR-MS has been used for mapping changes in lipid classes in the brain in the subventricular zone, a structure composed of several distinct cell layers, demonstrating how lipids play an important role in the regulation of adult neurogenesis^10^. MALDI has also been used to map lipids of the sebaceous gland and hair follicles^11^, lipid changes in the skin of leprosy patients^12^, and is used in cancer proteomics, neurology, ophthalmology, diabetes, cardiology, microbiology, and histopathology^13^.

For this study MALDI imaging with FT-ICR-MS was combined with Raman imaging to examine active DLE and clinically normal perilesional skin from the same patient. This combination provides a richer trove of information. Each technique extracts different but complimentary features of the biomolecules^14,15^, allowing a detailed analysis of the differences between DLE and control.

There were three objectives; firstly, to examine whether these techniques were technically feasible for the study of DLE-related biomolecules, secondly, to describe any significant differences between the involved and control samples to determine specific biochemical markers of DLE and thirdly, to understand any differences between the two that could explain the pathophysiology of DLE in greater detail.

## Methods

Detailed analysis instructions are included in the supporting information. All methods were carried out in accordance with relevant guidelines and regulations.

### Patient demographics

The participants were women with an average age of 48.5 years (standard deviation 13 years) and an average disease duration of 11 years (standard deviation 7 years). Their ethnicities were Samoan (two), Tongan and Māori. The biopsies were from the scalp (three) and the left preauricular region. A more detailed summary of demographics and treatment is in Supplementary Table S1.

### Tissue collection

Two 3 mm punch biopsies were taken from four patients with DLE. One of clinically involved active DLE and another of clinically normal skin to act as a control. Each biopsy was placed in 5% carboxymethylcellulose and immediately fresh frozen in an isopentane liquid nitrogen bath before being stored at − 80 °C until analysis. Informed consent was obtained from all subjects and/or their legal guardian(s).

The biopsies were sectioned using a Bright OTF5000 Cryostat (A-M Systems, USA) at − 20 °C and mounted on gold coated glass slides (Deposition Research Laboratory, Inc, USA).

The active DLE and the control sections were initially analysed using Raman hyperspectral imaging and then the same sections were analysed by MALDI FT-ICR-MS imaging.

### Raman spectroscopy and MALDI FT-ICR-MS imaging analysis

Sections were analysed using a Horiba LabRAM HR UV–VIS-NIR (Horiba, France) Raman microscope with a 785 nm laser. Samples were coated with 1,5-Diaminonapthalene deposited via sublimation, before subsequent MALDI FT-ICR-MS imaging analysis using a Bruker 7 T solarix-XR mass spectrometer (Bruker Daltonics, Bremen, Germany) in positive ion mode.

Raman spectrometry and MALDI data were processed and analysed using MatLabR2020b and HYPER-Tools^16^. Principal component analysis (PCA) of the imaging mass spectrometry data was undertaken to establish ions with a higher presence in DLE or control tissue.

### Peak assignments

PCA of the Raman data was undertaken to create a list of the key Raman bands (cm^-1^) that differentiate DLE from the control tissue and was analysed with reference to the comprehensive list of commonly observed Raman peaks in biological tissues and known biochemistry of the skin (Refer Table S2)^17–23^. The FT–ICR peaks that were identified as unique to the DLE epidermis by PCA (Table 1) had their peak assignments made by assessment of FT–ICR accurate masses and spatial distributions (Fig. S1), and database searching of accurate masses using LIPID MAPS^24^. The closest assignment within an error limit of 10 ppm was accepted, with manual exclusion of species of plant, fungal or prokaryotic origin. Where ≥ 2 assignments could not be discriminated, all possibilities are reported. It was not possible to perform tandem mass spectrometry using the MALDI-FT-ICR-MS directly from the tissue surface due to low abundance of the ions identified by PCA. Therefore, an integrated approach was used by investigating LC–MS/MS fragment ion spectra. When FT–ICR peaks were < 10 ppm error and also corresponded to analytes detected by LC–MS/MS with an error limit of < 10 ppm, the assignment made from the analysis of LC–MS/MS fragment spectra was accepted. When possible and to the best of our knowledge previously published reports identifying these assignments were also cited^25,26^. Under the Metabolomics Standards Initiative proposed by the Chemical Analysis Working Group, the analyte assignments in this study represent ‘putatively annotated compounds’^27^.

**Table 1:** Epidermal lipid profile unique to active Discoid Lupus Erythematosus compared to non-involved skin identified by Principal Component Analysis.

Lipid category	Lipid type	Observed m/z	Predicted m/z	FT-ICR -MS error (ppm)	Molecular formula	Identification	Ion	Validated by LC–MS/MS*	Previously observed in human skin
Confirmed assignments									
Sphingolipids	Sphingomyelins	703.5763	703.5748	2.1	C_39_H_79_N_2_O_6_P	SM 34:1;O2	[M + H] +	Y	(^11,25^)
Glycerophospholipids	Glycerophosphocholines	734.5739	734.5694	6.1	C_40_H_80_NO_8_P	PhC 32:0	[M + H] +	Y	(^11,25^)
		810.6035	810.6007	3.5	C_46_H_84_NO_8_P	PhC 38:4	[M + H] +	Y	(^11,25,28^)
		812.6127	812.6164	4.6	C_46_H_86_NO_8_P	PhC 38:3	[M + H] +	Y	(^29,30^)**
Assignments with two potential matches									
Sphingolipids	Sphingomyelins	*811.6131*	811.6090	5.1	C_44_H_89_N_2_O_6_PK	SM 39:1;O2	[M + K] +	–	(^31^)**
Glycerolipids	Triacylglycerides		811.6212	10.0	C_49_H_88_O_6_K	TG 46:3	[M + K] +	Y	(^28^)
Glycerolipids	Triacylglycerides	*891.6611*	891.6838	25.5	C_55_H_96_O_6_K	TG 52:5	[M + K] +	Y	(^28^)
Glycerophospholipids	Glycerophosphoinositols		891.6685	8.3	C_49_H_97_O_12_P	PhI O-40:0	[M + H-H_2_O] +	–	

Significant values are in italics.

*C* Carbon, *H* Hydrogen, *N* Nitrogen, *O* Oxygen, *P* Phosphorus, *Na* Sodium, *K* Potassium, *M* Molecule, *SM* Sphingomyelin, *PhC* Phospatidylcholine, *TG* Triacylglycerides, *m/z* mass-to-charge ratio. *LC–MS/MS* Liquid chromatography tandem mass spectrometry.

*The mass error limit was ≤ 10 ppm, refer to Table S3.

Error = difference between the observed m/z and the predicted m/z of the lipid molecule reported in parts per million (ppm).

**Previously reported in human plasma.

The study had ethics approval from the New Zealand Health and Disability Ethics Committee reference number 19/NTB/226.

### Statistical analysis

MALDI and Raman images generate large data sets. Principal component analysis is a multivariate statistical analysis tool that reduces the number of variables to only a few principal components but preserves the most relevant information. PCA finds the biggest variance in the dataset and assigns it to the first principal component (PC1), then finds the biggest variance in the remaining information and assigns it to a second component (PC2), and so on. Each principal component (PC) describes variance in the data that is independent of the other PCs. Only those PCs describing the relevant variance in the data are used to examine and create the molecular images, enabling exclusion of spurious signals such as instrument noise.

## Results

Four tissue sections (see supplementary Fig. S2) were processed by Raman spectroscopy (Patients 1,2,3 and 4) and three by mass spectrometry (Patients 1, 2, and 3). The tissue for patient 4 was not able to be analysed by mass spectrometry post Raman analysis due to delays imposed by Covid-19.

### Interpreting principal component analysis of Raman spectroscopy and mass spectrometry

**Figure 1:**
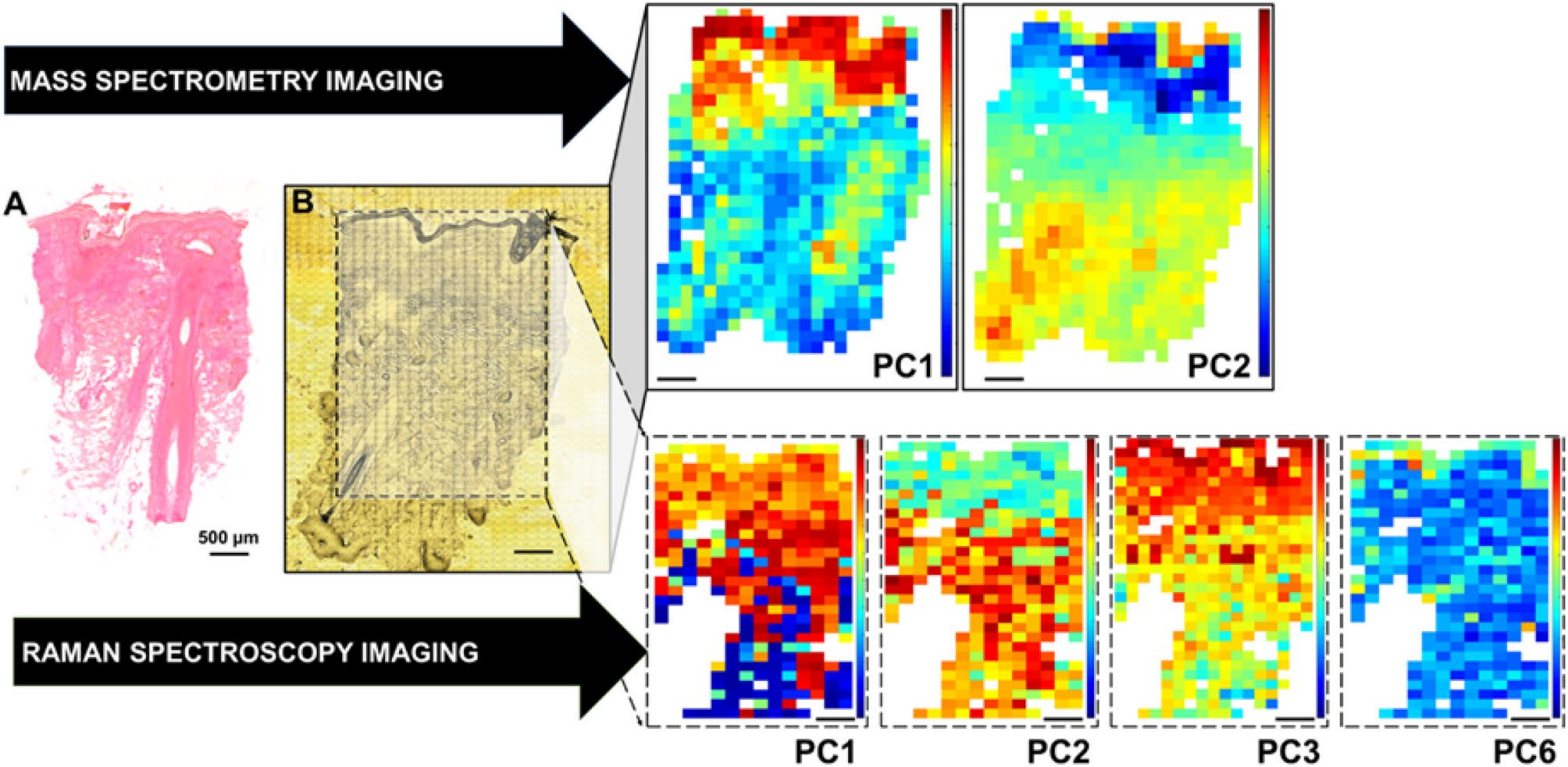
Sectioned discoid lupus erythematosus tissue viewed with (**A**) haematoxylin and eosin staining and (**B**) confocal microscopy and then subsequent heat maps with Mass Spectrometry and Raman Spectroscopy analysis. Each small grid visible in B represents individual targets for the mass and Raman spectroscopy analysis (Scale bar 500 μm). *PC* principal component.

**Figure 2:**
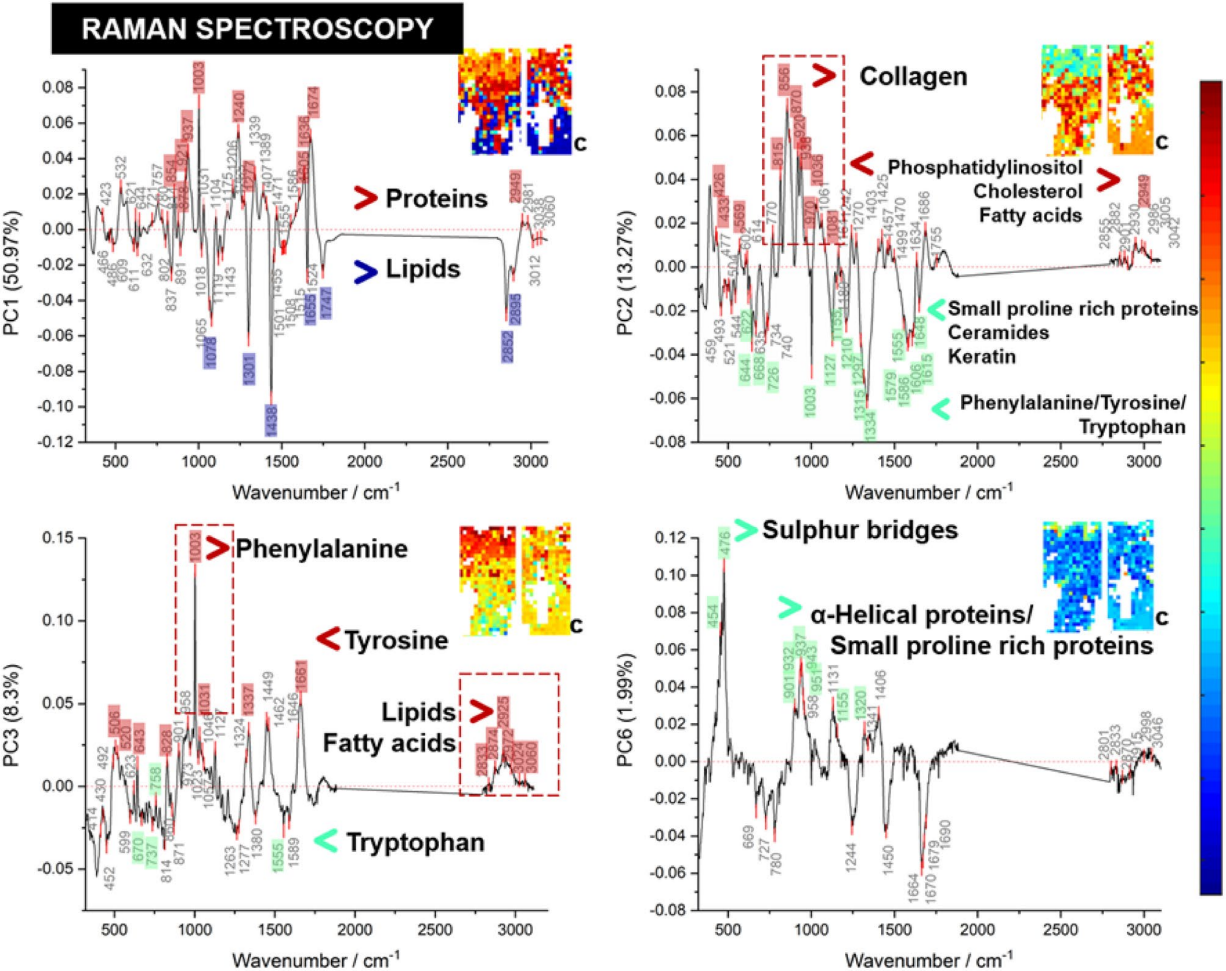
The images presented are the principal component analyses results contrasting all discoid lupus erythematosus images with control. The different principal components 1, 2, 3 and 6 displayed separately for clarity. Key findings are identified with arrows (< , >) to indicate the key molecules most abundant in either the positive or negative regions, *PC* Principal Component. *C* Control (Clinically normal perilesional skin).

**Figure 3:**
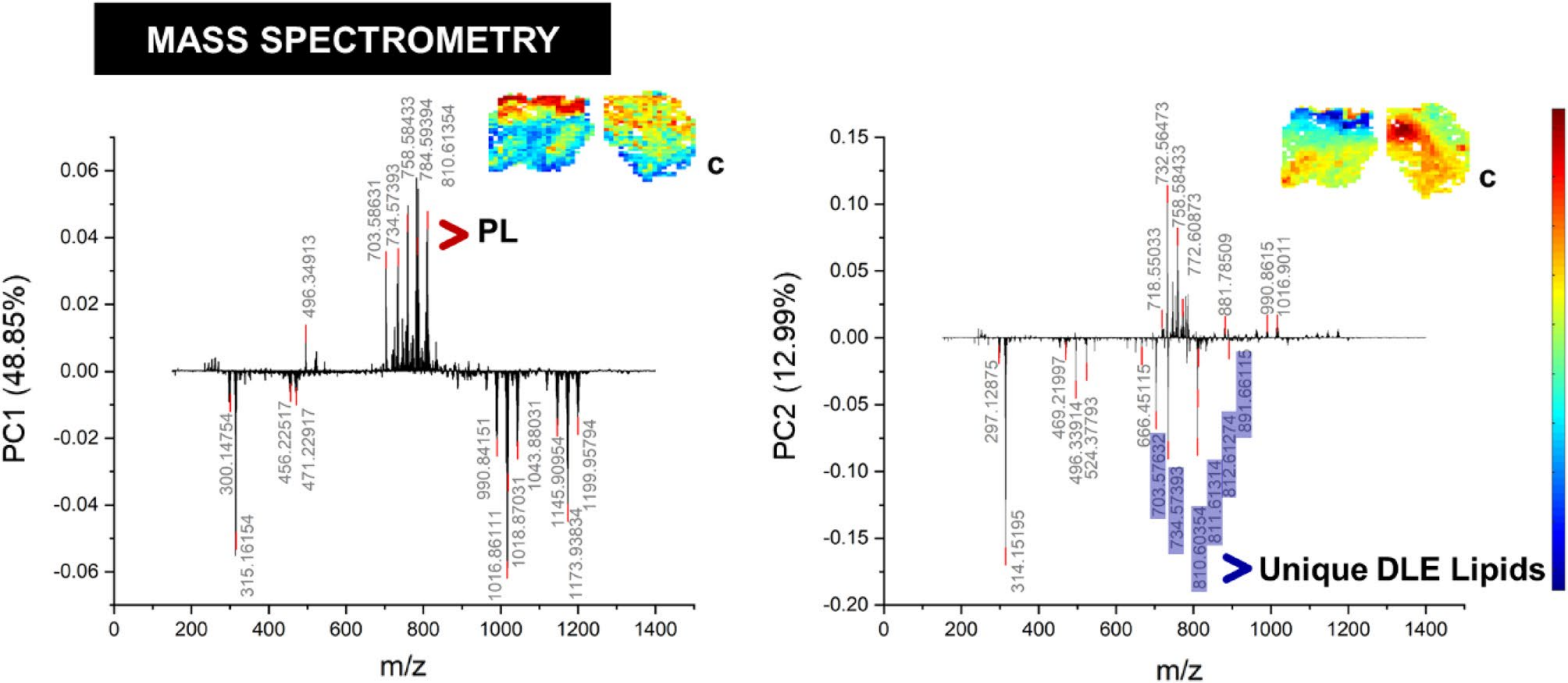
The mass spectrometry loadings with scores plots between DLE and control included as an inset. Arrows indicate which molecules are more abundant in either the positive or negative regions. *PC* = Principal Component, *C* Control (Clinically normal perilesional skin), *PL* Phospholipid, *DLE* Discoid Lupus Erythematosus.

Figure 1 summarises how a haematoxylin & eosin section (A) relates to the imaged section recorded by a confocal microscope (B). Areas of interest are selected in the imaged regions prior to analysis by Raman spectroscopy and imaging mass spectrometry. Each PC produces a heat map called a “scores plot”. The colours show the relative amounts of biomolecules in these areas (PC1 and PC2 for mass spectrometry and PC 1, 2, 3, and 6 for Raman spectroscopy). To identify which molecules are present the loadings plots are interpreted (Figs. 2 and 3), by referring to the positive (red) and negative (blue) peaks along the PC y axis. The top red colours in the legend indicate highest intensity for the positive loadings, and the bottom blue indicates the highest intensity for the negative loadings. The colours of the heat maps provide a useful comparison of which molecules are more or less present in DLE tissue relative to control. The score image and loadings plot for each PC provide unique information about the differences in biomolecules between the two.

The melanin content in hair exhibits strong background noise suppressing meaningful findings therefore background signal contributions from hair follicles in the Raman images have been masked (appearing white) for clarity.

### Raman spectroscopy imaging

Figure 2 shows the PCA loadings and associated score plot images (inset) for each of PC1, PC2, PC3 and PC6. The score plot images for all sections measured from all four patients comparing DLE and clinically normal perilesional skin is presented in Supplementary Fig. S3A–D. PC4 and PC5 did not display histologically meaningful clustering nor differences between the control and DLE tissue. The two score plot images in each inset show the DLE section (left image) and control labelled “C” (right image). The wavenumbers labelled on each “peak” in the loading plots represent the vibrational frequencies characteristic for specific biomolecules.

In PC1 the major source of variation is between the epidermis/dermis (red) and the subcutaneous fat (blue). The subcutaneous fat has a greater fat component than the epidermis and dermis shown by the strong and distinct lipid signals at 1078, 1301, 1438, 1655, 1747, 2582 and 2895 cm^−1^. This contrasts with the relatively higher protein content found in the keratin in the epidermis and collagen in the 854, 878, 921, 937, 1003, 1240, 1277, 1636 and 2949 cm^−1^ reflecting known histologic features.

The scores image plot of PC2 shows a green band demonstrating a thickened DLE epidermis absent in the control. The highlighted peaks from the negative loadings represent proteins including keratin (1155 and 1648 cm^−1^), ceramides (1127 and 1297 cm^−1^), both protein-bound and free aromatic amino acids phenylalanine, tyrosine involved in melanogenesis (622, 644,1003,1210 and 1555–1616 cm^−1^), and the C-S stretching bands (668 and 726 cm^−1^) of small proline-rich proteins which are known to form disulphide bonds or sulphur-sulphur linkages (S = S) with proteins including type II keratin chains in the epithelial surface. This agrees with studies that have demonstrated that these components are a part of the cornified cell envelope, which is a highly insoluble and extremely tough structure formed beneath the cell membrane during terminal differentiation of keratinocytes. Hyperkeratosis in the epidermis is observed clinically in DLE. The hyperkeratotic skin at the surface of DLE would be expected to have the highest concentration of small proline rich proteins, ceramides and keratin.

The red areas identified in the PC2 score plot are dominated by collagen (815–938, 1036, 1081 cm^−1^), and lipids (970, 1457, 1755, 2855 cm^−1^) including cholesterol (426, 433 and 614 cm^−1^), phosphatidylinositol (569 and 770 cm^−1^) and fatty acids (2949 cm^−1^).

The PC3 score plots further emphasise the differences in the epidermis of DLE to the control. There is again a signal contribution from the aromatic amino acids phenylalanine, tyrosine and collagen bands, while the negative loadings notably include tryptophan. Further distinction arises from lipid and fatty acid responses between 2833 and 2972 cm^−1^, which can be explained by the mass spectrometry results. Finally, the bands 506 and 520 cm^−1^ are assigned to disulphide bonds or sulphur-sulphur linkages (S = S), providing further evidence of cross-linking events by structural surface proteins.

In PC6 a green band in the location of the stratum corneum is distinct to DLE. There are key bands in the 420–480 cm^−1^ and 901–951 cm^−1^ region that can be assigned to both α-helical proteins including α-keratin, and the proline/hydroxyproline from the small proline-rich proteins and these findings are accounted for by the hyperkeratosis.

 Again, demonstrating how hyperkeratosis can be visualized in Raman spectroscopy. Refer to Supplementary Table S2 for full assignment bands.

### Mass spectrometry imaging

Figure 3 summarises the mass spectrometry findings of the MALDI images of the active DLE and control. The score plot images for all sections measured from three patients comparing DLE and clinically normal perilesional skin is presented in Supplementary Fig. S4A,B. The PC 1 score images are like the initial findings in Raman spectroscopy in PC1, meaning that the greatest source of variance is between the epidermis/dermis and the subcutaneous fat. The positive loadings show that there is an accumulation of phospholipids. These occur at higher levels in the epidermis in the DLE, unlike the control where they are distributed more equally and in smaller amounts throughout the upper dermis and epidermis.

The scores plot of PC2 shows that the epidermis of DLE is fully blue and distinct from that in the control. The loadings have identified a unique and distinctive grouping of lipids. An integrated assignment approach identified these as glycerophospholipids and sphingolipids listed in Table 1^11,25,28–31^. Sphingomyelins and phospholipids (specifically the phosphatidylcholine species) are known to be abundant and readily ionizable lipids in skin.

## Discussion

This is the first time DLE has been examined concurrently by Raman spectroscopy and mass spectrometry. The key finding is a unique profile of epidermal lipid biomarkers that defines active DLE from perilesional non-involved skin. Specific lipids from the MALDI FT-ICR-MS image analysis were identified by PC2, and these identities were evaluated by LC–MS/MS. Two key lipid categories include sphingolipids and glycerophospholipids, and of these classes, sphingomyelin, and phospholipids (with the latter exclusively phosphatidylcholine) were identified (Table 1). These lipids are key for membrane function and are known to be abundant and readily ionizable lipids in skin. The two signals identified by LC–MS/MS that most closely matched m/z 811.6131 and m/z 891.6611 observed by MALDI-FT-ICR IMS analysis were identified as TG 46:3 an TG 52:5, respectively (Table S3). However, the MALDI-FT-ICR m/z of these observed TGs exceed the < 10 ppm error limit. Further research is needed to determine if the lipid profile is present in other sub types of cutaneous lupus.

Therefore, the MALDI-FT-ICR signals (m/z 811.6131 and m/z 891.6611) could not be assigned as triacylglycerides, and remained unassigned. The current mismatch of these m/z may be a reflection of the mechanistic differences in ionsation between MALDI and electrospray ionisation. Furthermore, it has been reported that triacylglycerides are difficult to detect with MALDI, while the water soluble glycerophosphoinositols poses problems for conventional chromatographic protocols in terms of resolution and reproducibility, and these species may not be triacylglycerides^28,32^. Further work could elucidate these putative lipid identities. Looking at the data holisitically, further support for identification of sphingolipids over glycerolipids may be provided by the Raman Spectroscopy which demonstrates the increased presence of ceramide containing species in the epidermis. The Raman spectroscopy imaging results also demonstrate ceramides are associated with increased aromatic amino acids (tryptophan, phenylalanine, and tyrosine) and a lower lipid and fatty acid content. It is interesting to consider the potential significance of these findings.

Lipid biochemistry of the skin is highly complex. Lipids are not only essential for barrier function but also in the modulation of keratinocytes and the immunology of the skin. Lipid abnormalities are described in atopic dermatitis, psoriasis, rosacea, acne, various ichthyoses, allergic contact dermatitis and hidradenitis suppurativa^33^. Sphingolipids such as ceramide, sphingosine, sphingosine-1‑ phosphate (S1P), ceramide- phosphate and lyso-sphingomyelin, have roles in the regulation of cell growth, death, senescence, adhesion, migration, inflammation, angiogenesis and intracellular trafficking^34^.

Sphingomyelins, phospholipids and hexosylceramides are examples of bioactive membrane lipids, important in cell communication. In particular sphingomyelins and hexosylceramides are classes of sphingolipids, a ubiquitous constituent of biomembranes and include a large family of bioactive signaling molecules that are involved in several cellular processes including apoptosis, cell proliferation, migration and differentiation^34^. Their dysregulation has been described in various inflammatory and immune mediated diseases^35^. The role of lipids has been examined in systemic lupus erythematosus (SLE) and a significant correlation exists between the lipid diacyl phosphatidylethanolamine and systemic lupus activity measured by the SLE disease activity index (SLEDAI)^36^. There are a wide range of lipids differentially expressed in the serum of patients with SLE compared to those of healthy controls when examined by mass spectrometry identifying novel biomarkers including sphingolipids^37^. A mechanism of lipoapoptosis using palmitate activation by fatty acid transport protein 4 (FATP4) as a model described how increased neutral lipid content leads to an increase in the sphingolipids hexosylceramide and sphingomyelin as well as the phospholipid phosphatidylcholine ultimately leading to increased apoptosis^38^.

Lipid rafts are organised microdomains in the plasma membrane rich in lipids including cholesterol, glycosphingolipids, and sphingomyelin and these regions are involved in the signalling and activation of cells including T cells^39,40^. T cell signalling from these lipid rafts differs in patients with SLE^40,41^. CD4 positive T cells from SLE patients have an altered profile of lipid raft-associated glycosphingolipids. Lipid rafts are a potential therapeutic target^42^. Normalising glycerophospholipids has been shown to corrects CD4 + ve T cell signalling and functional defects as well as decreasing anti-double stranded DNA antibody production by autologous B cells in SLE patients^43^.

DLE is a photosensitive disorder with a majority reacting to both ultraviolet (UV) A and B or only UVB^44^. Ceramide is a key molecule in the sphingolipid pathway^45^. Therefore, it is interesting that increased de novo ceramide synthesis signals UVB-induced apoptosis in cultured normal human keratinocytes^46^.

The relevance of the epidermal accumulation of the amino acids phenylalanine, tyrosine and tryptophan is uncertain. Pigmentary change in DLE is common. The four participants in this study who were analysed all had skin of colour. Phenylalanine is converted to tyrosine which is essential for melanin synthesis. Phenylalanine accumulates in the epidermis of patients with vitiligo^47^. Tryptophan catabolism has a role in T cell apoptosis^48,49^. Furthermore, 6-formylindolo[3,2-b]carbazole (FICZ) a tryptophan photoproduct and endogenous high-affinity aryl hydrocarbon receptor (AhR) agonist, acts as a nanomolar photosensitizer potentiating UVA-induced oxidative stress^50^. Additionally, the subsequent generation of reactive oxygen species (ROS), responsible for UV photodamage, occurs through various mechanisms including UV-enhanced electron leakage from the mitochondrial respiratory chain and remodelling of cholesterol-rich plasma membrane rafts of keratinocytes^51,52^.

The Raman spectroscopy imaging results match DLE histology stained by haematoxylin and eosin. For example, hyperkeratosis in the stratum corneum and increased dermal collagen, findings that are consistent with current knowledge of DLE. It is reassuring that these expected findings are confirmed by this methodology and therefore support the veracity of the additional findings of abnormalities in lipids and amino acids.

Furthermore, the study demonstrates that there is a future potential theoretical application of these techniques through a combinatorial use of artificial intelligence combining Raman and mass spectrometry analysis of skin disease to assign a diagnosis based on the molecular composition of the sample rather than the histological appearance^53^. Stimulated Raman histology is used to provide real time histology perioperatively particularly in the field of neurosurgery^54^.

Raman and mass spectrometry is a viable investigational tool in DLE and could be applied to other skin diseases. The study has demonstrated new findings of a distinctive lipid and protein profile in active DLE with increased aromatic amino acid composition. It is intriguing to speculate on the role of disordered lipid biochemistry, lipid rafts, cell signalling and amino acids in the pathophysiology of DLE.

### Supplementary Information

Supplementary Information.

## Data Availability

This dataset will be described and assigned a citable DOI via the institutional data publishing platform—https://auckland.figshare.com/. An embargo will be in place whilst papers derived from the dataset are prepared and published. During the embargo period, dataset access will be mediated by Dr Hannah Holtkamp, lead author, following details provided in the published description of the dataset. Conditions for access and use will be set out in data sharing agreement, commonly between institutions. The dataset will be published for public access at the end of the embargo period.
