# Supplementary material for: Raman spectroscopy and mass spectrometry identifies a unique group of epidermal lipids in active discoid lupus erythematosus
Source: Sci Rep. 2023 Sep 30;13:16452. doi: 10.1038/s41598-023-43331-3 (PMC10542761; doi:10.1038/s41598-023-43331-3)
Supplement: Supplementary file 1 — Supplementary Information. [file 41598_2023_43331_MOESM1_ESM.docx]

**Supporting Information** to:

Raman spectroscopy and mass spectrometry identifies a unique group of epidermal lipids in active discoid lupus erythematosus.

**Methods**

*Tissue Processing*

All tissue sections were cut transversely and dehydrated in a vacuum desiccator (Jencons, USA) for at least 1 hour.

*Raman Spectroscopy Imaging*

Raman spectra were collected using the Horiba LabRAM HR UV-VIS-NIR (Horiba, France) Raman microscope. A 785 nm laser with 24 mW power and a long focal distance microscope objective 50x (Olympus) was used. The confocal pinhole was set to 200 μm. The collected light was filtered through an edge filter and dispersed with a 4 cm^−1^ spectral resolution, using a grating of 600 grooves per mm over a wavenumber range of 200–3200 cm^−1^. Spectral acquisition and instrument operation was done using LabSpec 6 (Horiba, France). In all mapping measurements, an m × n square was placed to include the entirety of tissue from stratum corneum until fatty tissue identified by the confocal microscope, with a step size of 150 µm. For each spectrum a 40 s exposure time was used with 3 accumulations.

Assignments were made by searching the table of commonly observed Raman peaks in biological tissue reported by Rehman et al (1).

*MALDI-MS Imaging*

The samples were rinsed with ammonium formate (50 mM) (2) to increase the signal intensity and the signal-to-noise ratio. The samples were coated with 1,5-Diaminonapthalene (Sigma-Aldrich, St Louis, MO) deposited *via* sublimation using the methods described by Chaurand et al (3) using an in-house vacuum sublimation apparatus for 5 mins at approximately 50 mTorr and 140°C.

MALDI Imaging analysis was carried out using a Bruker 7 T solarix-XR mass spectrometer (Bruker Daltonics, Bremen, Germany) with MALDI ionisation at 50 µm spatial resolution. Spectra were collected in the m/z range of 150-2000. FT-ICR data were exported in flexImaging v4.1 (Bruker Daltonics, Bremen, Germany) and normalised using the root mean square method.

Assignments were made by database searching of accurate masses using LIPID MAPS (4). Pre-processing, and principal component analysis was performed using Matlab® R2020b (Math Works, Natick, MA, USA) and HYPER-Tools v.3.0 (5).

*Liquid Chromatography with Tandem Mass Spectrometry*

Methanol and propanol (Optima LC-MS grade) were obtained from Thermo Fisher Scientific (Auckland, Aotearoa New Zealand). Type 1 water was generated from a Merck Millipore unit (Auckland, Aotearoa New Zealand). AnalaR reagents included glacial acetic acid [GAA], also from Merck Millipore, and butanol, chloroform and ammonium hydroxide from Sigma-Aldrich Pty Ltd (Sydney, Australia). The SPLASH Lipidomix solution containing isotopically labelled internal standards was acquired from Avanti Polar Lipids, Inc (Alabama, USA).

Frozen tissue sections were sectioned from all four patient cases for lipid analysis and identification by liquid chromatography–tandem mass spectrometry (LC–MS/MS). Tissue was transferred cold into glass amber vials and weighed. Lipid extraction was performed in the following solvent mixture water:butanol:methanol:chloroform:SPLASH at a ratio of 4:15:15:20:1, respectively. Samples were homogenised with a Potter tissue homogeniser and sonicated for 30 minutes. The samples were then centrifuged at 6000 g for 10 minutes at 4°C. The supernatant was transferred to a fresh amber autosampler vial with microinsert using a glass Pasteur pipette, capped, and loaded into the LC-MS/MS analysis.

The liquid chromatography comprised an Accela 1250 pump (Thermo Fisher Scientific, Waltham, MA, USA), a Thermo TriPlus autosampler (Thermo Fisher Scientific, Hampton, VA, USA), a HotDog5090 column oven and a Kinetex F5 analytical column (100 mm × 2.1 mm × 2.6 µm) (Phenomenex Inc., California, USA). The column and samples were maintained at temperatures of 45°C and 20°C , respectively. Injection volume was 5 μL.

Mobile phases were water + 0.05% acetic acid + 5 mM ammonium (Solvent A), methanol + 0.05% acetic acid + 5 mM ammonium (Solvent B) and isopropanol + 0.05% acetic acid + 5 mM ammonium (Solvent C). Reversed-phase LC was conducted at a flow rate of 0.4 mL min^-1^ was applied with a gradient elution described below:

| Minutes | %A | %B | %C |
| --- | --- | --- | --- |
| 0 | 30.00 | 70.00 | - |
| 5 | 5.00 | 95.00 | - |
| 14.65 | 5.00 | 25.00 | 70.00 |
| 14.75 | 5.00 | 5.00 | 90.00 |
| 15.5 | 5.00 | 5.00 | 90.00 |
| 16 | 5.00 | 95.00 | - |
| 16.5 | 30.00 | 70.00 | - |
| 20 | 30.00 | 70.00 | - |

The mass spectrometer was a Q-Exactive (Thermo Scientific, Dreieich, Germany) with a heated electrospray ionisation [ESI] source. Source voltage was 3.5 kV, S-lens RF level 55, heated capillary temperature 375 ◦C, sheath gas flow rate of 40 units, auxiliary gas flow rate of 8 units, sweep gas flow rate of 0 units, and a max spray current of 100 µA. Quantitative and qualitative mass spectral data was acquired using Data Dependent Analysis [DDA]. For positive ionisation the full scan range was m/z 200-1,250. Two precursors were selected for MS/MS scans every cycle with AGC 1e5, max IT time of 100 ms, isolation window of 2 m/z and collision energy of 25.

Data was processed using MS-DIAL v4.92 (Tsugawa et al. 2015, Tsugawa et al. 2020). The results were manually filtered to remove false positives and noise features.

**Results**


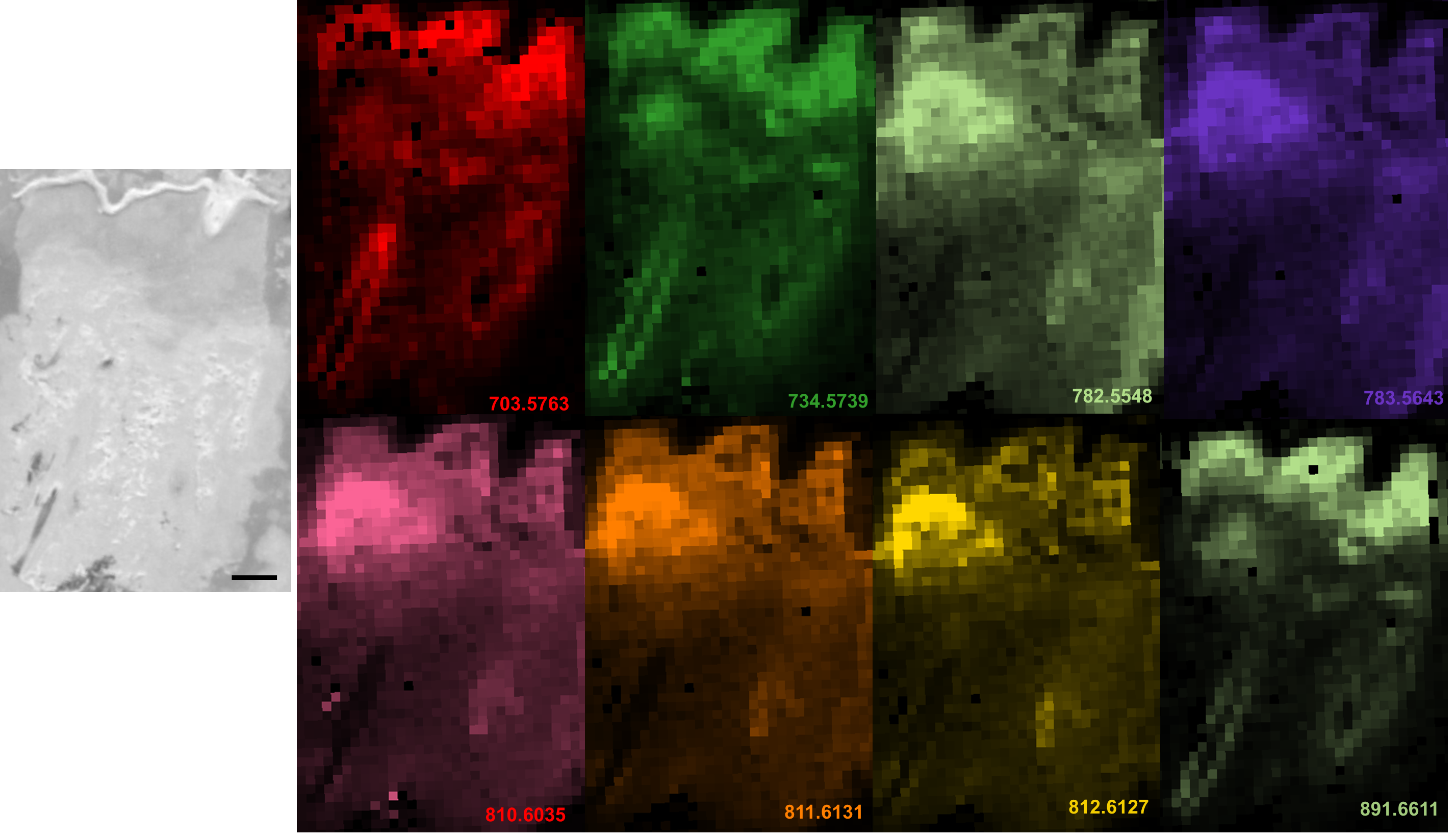


Figure S1. The mass-to-charge (m/z) image maps of the lipids identified in Table 2. Showing that there is an accumulation of lipids in the epidermis of the DLE tissue.


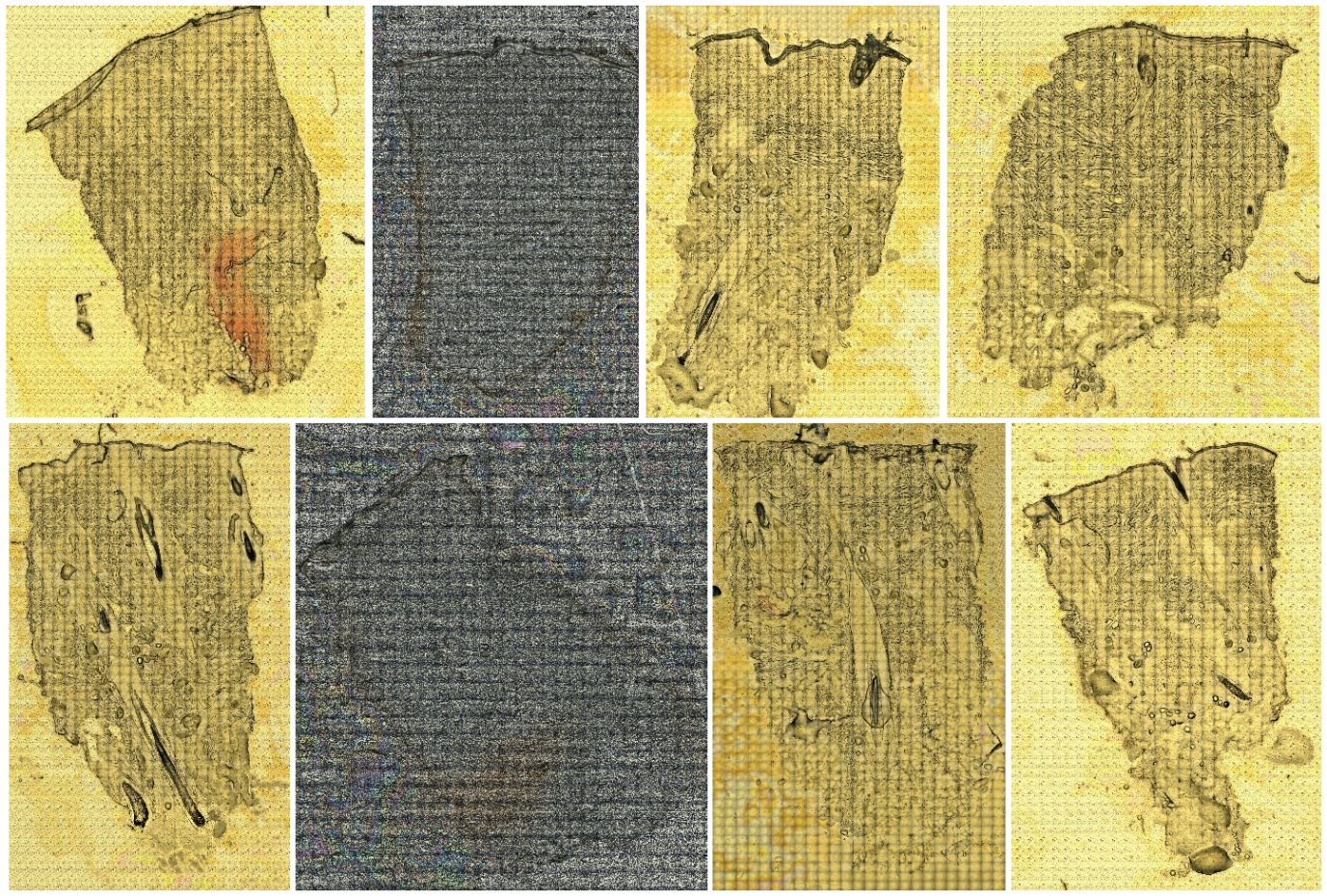


**C**

**C**

**C**

**C**

Figure S2. The images of 20 µm sectioned DLE and clinically normal perilesional skin (c), captured through the Horiba Raman confocal microscope.


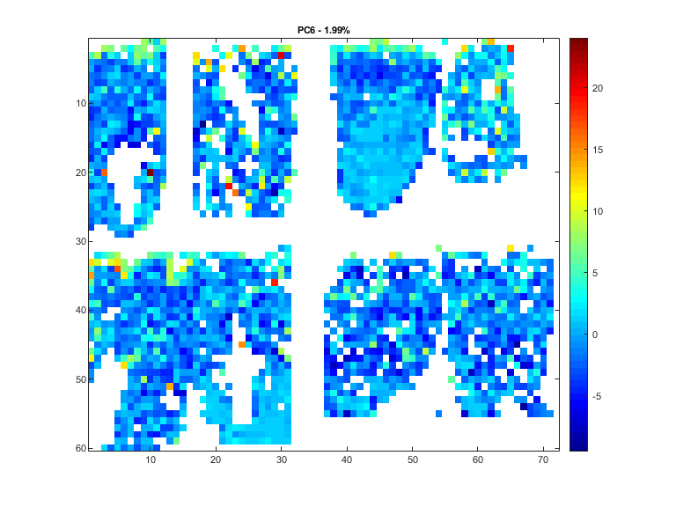

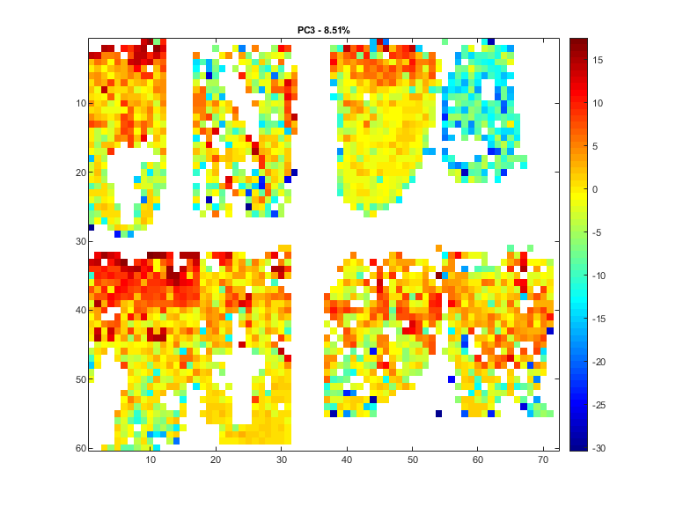

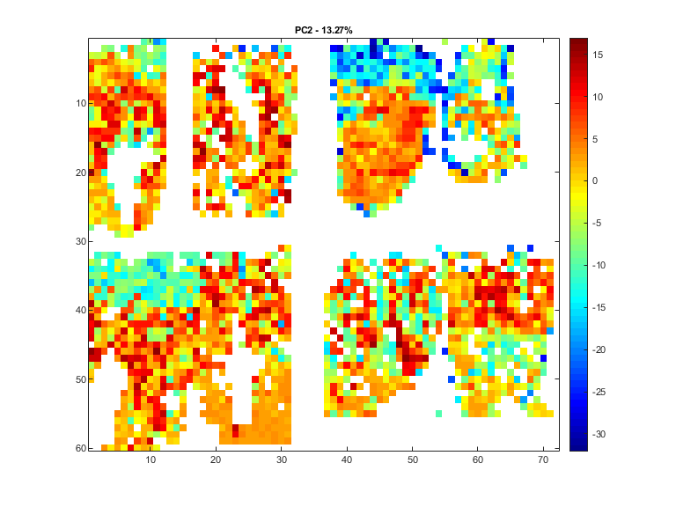

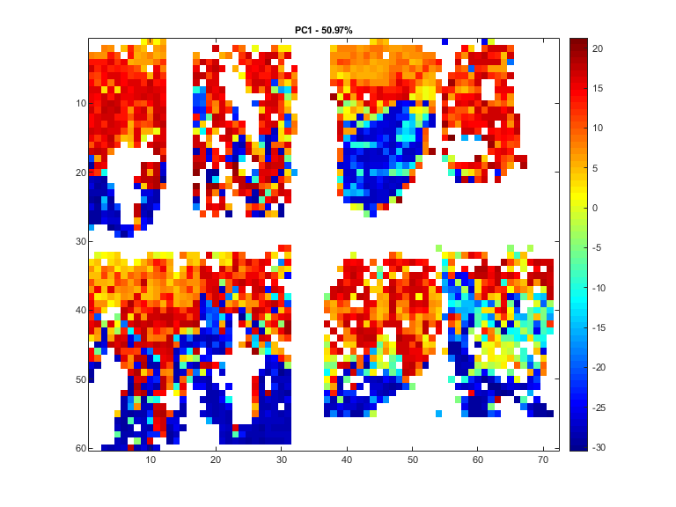


**A**

**B**

**C**

**D**

C

C

C

C

C

C

C

C

C

C

C

C

C

C

C

C

Figure S3 (A-D). The complete principal component analysis score plots of all Raman spectroscopy imaging data of all sections from all four patients (DLE (left) verses clinically normal perilesional skin (right, indicated by c) used to generate the loadings.


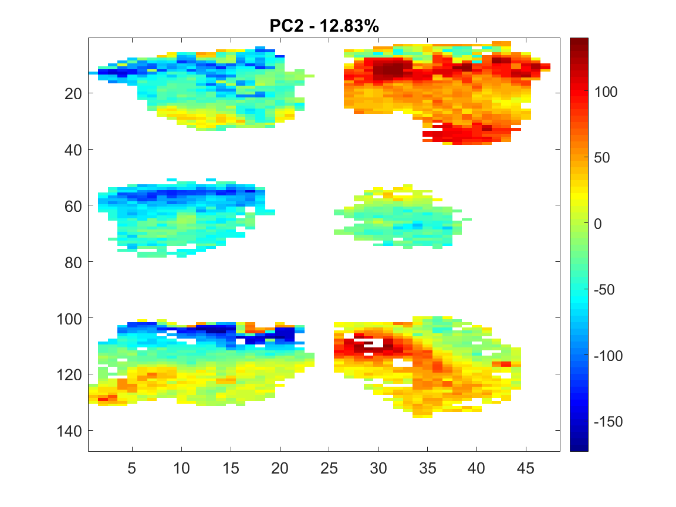

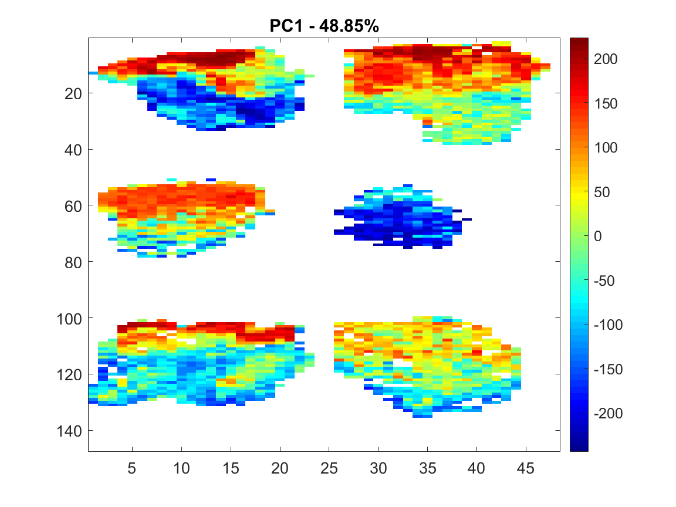


**A**

**B**

C

C

C

C

C

C

Figure S4(A-B). The complete principal component analysis score plots of all mass spectrometry imaging data of sections from three patients (DLE (left) verses clinically normal perilesional skin (right, indicated by c) used to generate the loadings.

Table S1. Demographics and previous treatments of participants.

| **Gender** | **Age (years)** | **Approximate Disease duration (years)** | **Previous treatments** | **Biopsy site** |
| --- | --- | --- | --- | --- |
| Female | 54 | 19 | CP, HQ, Pred, Iso, Dap, MTX, Clo, Myco, Tac | Scalp |
| Female | 37 | 12 | CP, Mom, HQ, Pred | L pre auricular |
| Female | 64 | 2 | CP, HQ, | Scalp |
| Female | 38 | 10 | CP, HQ, Myco | Scalp |

CP=Clobetasol propionate (ultrapotent topical steroid) HQ=Hydroxychloroquine, Pred=Prednisone, Iso=Isotretinoin, Dap=Dapsone, MTX=Methotrexate, Clo=Clofazamine, Myco=Mycophenolate mofetil, Tac=Topical tacrolimus

Table S2 The assignment of Raman bands identified by the PCA analysis of DLE and unaffected adjacent control tissue. The principal peaks included in the results and discussion have an Asterix by the wavenumber. Unless stated otherwise, assignments were made referencing "Raman Spectroscopy of Biological Tissues" (1).

| PC1 | 423 | Cholesterol, cholesterol ester |
| --- | --- | --- |
| Positive | 532 | Cholesterol ester |
|  | 621 | C‒C twisting mode of phenylalanine (proteins) |
|  | 644 | C‒C twisting mode of tyrosine |
|  | 721 | C‒C stretching, α-helix (proteins) |
|  | 757 | Tryptophan |
|  | 780 | Uracil-based ring breathing mode |
|  | 814 | C‒C stretching (collagen assignment) |
|  | 854* | Ring breathing tyrosine (proteins) |
|  | 878* | Hydroxyproline, tryptophan |
|  | 921* | Proline ring/glucose/lactic acid |
|  | 937* | C-C backbone, proline (collagen); C-C stretching α-helix (proteins); C-O-C glycosides (carbohydrates) |
|  | 1003* | Phenylalanine, C‒C skeletal |
|  | 1031 | δ(C‒H), phenylalanine (protein assignment), C‒N stretching of proteins, Carbohydrate residues of collagen |
|  | 1104 | Phenylalanine (proteins) |
|  | 1175 | Tyrosine, phenylalanine, C‒H bend (protein) |
|  | 1206 | Hydroxyproline, tyrosine (collagen assignment) |
|  | 1240* | Collagen |
|  | 1265 | Amide III (collagen assignment) |
|  | 1277* | Proteins, including collagen I |
|  | 1339 | CH2/CH3 wagging and twisting mode in collagen, nucleic acid and tryptophan |
|  | 1407 | ν_s_ COO^−^ (IgG?) |
|  | 1471 | C=N stretching |
|  | 1555 | Amide II |
|  | 1586 | Phenylalanine, hydroxyproline |
|  | 1605 | Phenylalanine, tyrosine, C˭C (protein) |
|  | 1636* | Amide I band (both α-helix and β-structure), collagen |
|  | 1674* | C˭C stretch vibration, cholesterol |
|  | 2949 | C‒H vibrations in lipids and proteins |
|  | 2981 | cholesterol ester |
| PC1 | 466 | Polysaccharides (amylase, amylopectin) |
| Negative | 486 | Glycogen |
|  | 609 | Cholesterol |
|  | 611 | Cholesterol ester |
|  | 632 | ν(C-S) gauche (amino acid methionine) |
|  | 802 | Uracil-based ring breathing mode |
|  | 837 | Deformative vibrations of amine groups |
|  | 891 | Saccharide band (overlaps with acyl band) |
|  | 1018 | Stretching C‒O ribose |
|  | 1065 | Skeletal C‒C stretch of lipids |
|  | 1078 | ν(C‒C) or ν(C‒O), phospholipids (lipid assignment) |
|  | 1119 | C‒C stretch (breast lipid) |
|  | 1143 | C‒C skeletal stretching |
|  | 1301* | Lipid |
|  | 1438* | Lipid |
|  | 1455 | Collagen and phospholipids |
|  | 1501 | C‒C stretching in benzenoid ring |
|  | 1508 | N-H bending |
|  | 1515 | Cytosine |
|  | 1524 | In-plane vibrations of the conjugated -C˭C- |
|  | 1655 | Lipid |
|  | 1747 | C˭O, lipids |
|  | 2582* | C‒H stretches |
|  | 2895* | CH_3_ symmetric stretch |
|  | 3012 | Unsaturated =CH stretch |
| PC2 | 426 | Cholesterol, cholesterol ester |
| positive | 433 | Cholesterol, cholesterol ester |
|  | 569 | Phosphatidylinositol |
|  | 602 | Phosphatidylinositol |
|  | 614 | Cholesterol ester |
|  | 770 | Phosphatidylinositol |
|  | 815 | Proline, hydroxyproline, tyrosine, ν_2_ PO_2_^−^ stretch of nucleic acids |
|  | 856* | Amino acid side chain vibrations of proline and hydroxyproline, as well as a (C‒C) vibration of the collagen backbone hydroxyproline (collagen type I) |
|  | 870* | C‒C stretching, hydroxyproline (collagen assignment) |
|  | 920* | C‒C, proline ring (collagen assignment) |
|  | 938* | Proline, hydroxyproline, ν(C‒C) skeletal of collagen backbone |
|  | 967 | Lipids |
|  | 970 | Phosphate monoester groups of phosphorylated proteins and cellular nucleic acids |
|  | 1036 | Collagen |
|  | 1061 | C‒C in-plane bending |
|  | 1081 | Collagen, Tryptophan |
|  | 1164 | Tyrosine (collagen type I) |
|  | 1242 | Amide III (β sheet and random coils) |
|  | 1270 | Amide III band in proteins, traditionally been attributed to amide III, a C‒N stretch from alpha helix proteins |
|  | 1403 | Bending modes of methyl groups (one of vibrational modes of collagen) |
|  | 1425 | CH_2_ deformation (lipid)/ NH in-plane deformation |
|  | 1457 | CH_2_/CH_3_ deformation of lipids and collagen |
|  | 1470 | Lipids |
|  | 1499 | C‒C stretching in benzenoid ring |
|  | 1634 | Amide I |
|  | 1686 | Amide I (disordered structure; non-hydrogen bonded) |
|  | 1755 | C˭O (lipid) |
|  | 2855 | CH_2_ symmetric stretch of lipids |
|  | 2882 | CH_2_ asymmetric stretch of lipids and proteins |
|  | 2901 | CH stretch |
|  | 2930 | CH_2_ asymmetric stretch |
|  | 2949 | ν_as_ CH_2_, lipids, fatty acids |
|  | 2986 | cholesterol ester |
|  | 3005 | Bond ‒C‒H stretch |
|  | 3042 | ν=CH of lipids |
| PC2 | 459 | Ring torsion of phenyl (2) |
| negative | 493 | Glycogen |
|  | 544 | Cholesterol |
|  | 622* | C‒C twisting mode of phenylalanine (proteins) |
|  | 635 | Tyrosine ring breathing |
|  | 644* | C‒C twisting mode of phenylalanine (proteins) |
|  | 668 | C-S stretching |
|  | 726 | C-S (protein) |
|  | 734 | Phosphatidylserine |
|  | 740 | Tryptophan |
|  | 1003* | Phenylalanine, C‒C skeletal |
|  | 1127* | Ceramide |
|  | 1155 | C‒C (and C‒N) stretching of proteins (also carotenoids), glycogen |
|  | 1173 | Tyrosine (collagen type I) |
|  | 1180 | C‒H bending tyrosine (proteins); |
|  | 1210* | C‒C_6_H_5_ stretching mode in tyrosine and phenylalanine |
|  | 1297* | Ceramide |
|  | 1315* | C‒H deformation (protein) CH_3_CH_2_ twisting mode proline/hydroxyproline containing proteins |
|  | 1334* | CH_3_CH_2_ wagging proline/hydroxyproline containing proteins |
|  | 1555 | Tryptophan, ν (CN) and δ (NH) amide II (protein assignment), ν(C˭C) porphyrin, Tyrosine, amide II, COO− |
|  | 1579 | C˭C bending mode of phenylalanine |
|  | 1586 | Phenylalanine, hydroxyproline |
|  | 1606 | Phenylalanine, tyrosine, C˭C (protein) |
|  | 1615 | Tyrosine, tryptophan, C˭C (protein) |
|  | 1648 | Proteins, Random coils, Amide I (C=C) |
| PC3 | 492 | Glycogen |
| positive | 506 | S-S disulfide stretching band of collagen |
|  | 520 | S-S disulfide stretching in proteins; Phosphatidylserine |
|  | 623 | C‒C twisting mode of phenylalanine (proteins) |
|  | 643* | C‒C twisting mode of tyrosine |
|  | 828* | Proline, hydroxyproline, tyrosine |
|  | 901 | C‒C skeletal stretching |
|  | 958 | Hydroxyapatite, carotenoid, cholesterol |
|  | 973 | C‒C backbone (collagen assignment) |
|  | 1003* | Phenylalanine, C‒C skeletal |
|  | 1023 | Glycogen |
|  | 1031* | δ(C‒H), phenylalanine (protein assignment) |
|  | 1046 | Proline (collagen) |
|  | 1057 | Lipids |
|  | 1127 | ν(C‒N) |
|  | 1324 | CH_3_CH_2_ wagging mode present in collagen |
|  | 1337* | Amide III and CH_2_ wagging vibrations from glycine backbone and proline side chain |
|  | 1449 | C‒H vibration (proteins and lipids) |
|  | 1462 | CH_2_/CH_3_ deformation of lipids and collagen |
|  | 1646 | Proteins, Random coils, Amide I (α-helix) |
|  | 1661* | Amide I (structural proteins), Ceramide, ν(C˭C) cis, lipids, fatty |
|  | 2833 | CH_2_ symmetric stretch of lipids |
|  | 2874* | ν_s_ CH_3_, lipids, fatty acids |
|  | 2925 | CH stretch of lipids and proteins |
|  | 2972* | ν_as_ CH_3_, lipids, fatty acids |
|  | 3024 | ν=CH of lipids |
| PC3 | 414 | Phosphatidylinositol |
| negative | 430 | Cholesterol, cholesterol ester |
|  | 452 | Ring torsion of phenyl (2) |
|  | 599 | Phosphatidylinositol |
|  | 670* | Ring breathing of tryptophan |
|  | 737 | Tryptophan |
|  | 758* | Tryptophan |
|  | 870 | Most probably due to single bond stretching vibrations for the amino acids proline and valine and polysaccharides; C‒C stretching, hydroxyproline (collagen assignment) |
|  | 1263 | Lipids |
|  | 1277 | Proteins, including collagen I |
|  | 1380 | δCH_3_ symmetric (lipid assignment) |
|  | 1555* | Tryptophan |
| PC6 | 454 | Ring torsion of phenyl (2) |
| Positive | 476 | S-S bridges from reference (6). |
|  | 901 | C‒C skeletal stretching |
|  | 932* | Skeletal C‒C, α-helix, C‒C stretching mode of proline and valine and protein backbone (α-helix conformation) |
|  | 937* | C‒C stretching, α-helix (proteins), amino acid side chain vibrations of proline and hydroxyproline |
|  | 943 | Skeletal modes |
|  | 951* | ν_s_ (CH_3_) of proteins (α-helix) |
|  | 958 | Hydroxyapatite, carotenoid, cholesterol |
|  | 1131 | Phenylalanine, C‒N stretching of proteins |
|  | 1155 | C‒C (and C‒N) stretching of proteins (also carotenoids), glycogen |
|  | 1320 | CH deformation (proteins) |
|  | 1341 | CH deformation (proteins and carbohydrates) |
|  | 1406 | ν_s_ COO^−^ (IgG?) |
|  | 2801 | Contributions from acyl chains |
|  | 2833 | CH_2_ symmetric stretch of lipids (suggests change in the amount of lipid) |
|  | 2870 | CH_2_ asymmetric stretch and CH stretch of lipids and proteins |
|  | 2915 | CH stretch of lipids and proteins |
|  | 2998 | CH stretching |
|  | 3046 | ν=CH of lipids |
| PC6 | 727 | Lipid |
| negative | 780 | Phosphatidylinositol |
|  | 1244 | Amide III |
|  | 1450 | C‒H deformation bands (CH functional groups in lipids, amino acid side chains of the proteins and carbohydrates) |
|  | 1664 | Amide I |
|  | 1670 | Amide I |
|  | 1679 | Amide I (β-sheet) |
|  | 1690 | Amide I (disordered structure; non-hydrogen bonded) |

Table S3

3. The liquid chromatography tandem mass spectrometry analysis of the lipid peaks identified as most abundant in DLE epidermis by the PCA analysis of MALDI-MS imaging results.

| **ISTD** | **Alignment ID** | **Average RT (min)** | **Average m/z** | **Metabolite name** | **Ion** | **Molecular formula** | **Nominal** | **ppm** |
| --- | --- | --- | --- | --- | --- | --- | --- | --- |
| 9650 | 8831 | 6.467 | 703.57452 | SM 34:1;2O\|SM 21:0;2O/13:1 | [M+H]+ | C_39_H_79_N_2_O_6_P | 703.5754 | 1.25 |
| 11953 | 11523 | 10.367 | 811.62061 | TG 46:3\|TG 12:0_16:1_18:2 | [M+K]+ | C_49_H_88_O_6_ | 811.6218 | 1.46 |
| 10013 | 9537 | 8.373 | 734.56958 | PC 32:0\|PC 16:0_16:0 | [M+H]+ | C_40_H_80_NO_8_P | 734.57 | 0.54 |
| 10013 | 11491 | 8.493 | 810.60107 | PC 38:4 | [M+H]+ | C_46_H_84_NO_8_P | 810.6013 | 0.26 |
| 10013 | 11553 | 8.948 | 812.61566 | PC 38:3 | [M+H]+ | C_46_H_86_NO_8_P | 812.6169 | 1.56 |
| 11953 | 13378 | 10.973 | 891.67834 | TG 52:5\|TG 16:0_18:2_18:3 | [M+K]+ | C_55_H_96_O_6_ | 891.6844 | 6.80 |

Additional References:

1. A. C. S. Talari, Z. Movasaghi, S. Rehman, I. u. Rehman, Raman Spectroscopy of Biological Tissues. *Applied Spectroscopy Reviews* **50**, 46-111 (2015).

2. T. C. Baker, J. Han, C. H. Borchers, Recent advancements in matrix-assisted laser desorption/ionization mass spectrometry imaging. *Curr. Opin. Biotechnol.* **43**, 62-69 (2017).

3. A. Thomas, J. L. Charbonneau, E. Fournaise, P. Chaurand, Sublimation of New Matrix Candidates for High Spatial Resolution Imaging Mass Spectrometry of Lipids: Enhanced Information in Both Positive and Negative Polarities after 1,5-Diaminonapthalene Deposition. *Anal. Chem.* **84**, 2048-2054 (2012).

4. M. Marchetti-Deschmann, G. Allmaier, Allergenic compounds on the inner and outer surfaces of natural latex gloves: MALDI mass spectrometry and imaging of proteinous allergens. *J. Mass Spectrom.* **44**, 61-70 (2009).

5. J. M. Amigo, H. Babamoradi, S. Elcoroaristizabal, Hyperspectral image analysis. A tutorial. *Anal. Chim. Acta* **896**, 34-51 (2015).

6. S. G. Penteado *et al.*, Diagnosis of degenerative lesions of supraspinatus rotator cuff tendons by Fourier transform-Raman spectroscopy. *Journal of Biomedical Optics* **13**, 014018 (2008).
